# Supplementary material for: Altered expression of genes regulating inflammation and synaptogenesis during regrowth of afferent neurons to cochlear hair cells
Source: PLoS One. 2020 Oct 1;15(10):e0238578. doi: 10.1371/journal.pone.0238578 (PMC7529247; doi:10.1371/journal.pone.0238578)
Supplement: S1 Table — (DOCX) [file pone.0238578.s001.docx]

| **x-axis** |
| --- |
| OC_5h_KA_4 |
| OC_24h_CTRL_4 |
| OC_24h_KA_4 |
| OC_5h_CTRL_3 |
| OC_5h_KA_3 |
| OC_5h_CTRL_1 |
| OC_5h_KA_1 |
| OC_5h_KA_2 |
| OC_5h_CTRL_2 |
| OC_24h_KA_3 |
| OC_24h_CTRL_3 |
| OC_24h_CTRL_2 |
| OC_24h_CTRL_1 |
| OC_72h_KA_3 |
| OC_72h_CTRL_3 |
| OC_72h_KA_4 |
| OC_72h_CTRL_4 |
| OC_72h_KA_2 |
| OC_72h_CTRL_2 |
| SG_72h_CTRL_2 |
| SG_72h_KA_3 |
| SG_72h_CTRL_4 |
| SG_72h_KA_4 |
| SG_72h_KA_1 |
| SG_72h_CTRL_3 |
| SG_24h_CTRL_2 |
| SG_24h_KA_1 |
| SG_24h_CTRL_3 |
| SG_24h_KA_3 |
| SG_24h_KA_4 |
| SG_5h_CTRL_4 |
| SG_5h_KA_4 |
| SG_5h_KA_2 |
| SG_5h_CTRL_2 |
| SG_5h_KA_3 |
| SG_5h_CTRL_3 |
| SG_5h_KA_1 |
| SG_5h_CTRL_1 |

| **y-axis** |
| --- |
| SG_5h_CTRL_1 |
| SG_5h_KA_1 |
| SG_5h_CTRL_3 |
| SG_5h_KA_3 |
| SG_5h_CTRL_2 |
| SG_5h_KA_2 |
| SG_5h_KA_4 |
| SG_5h_CTRL_4 |
| SG_24h_KA_4 |
| SG_24h_KA_3 |
| SG_24h_CTRL_3 |
| SG_24h_KA_1 |
| SG_24h_CTRL_2 |
| SG_72h_CTRL_3 |
| SG_72h_KA_1 |
| SG_72h_KA_4 |
| SG_72h_CTRL_4 |
| SG_72h_KA_3 |
| SG_72h_CTRL_2 |
| OC_72h_CTRL_2 |
| OC_72h_KA_2 |
| OC_72h_CTRL_4 |
| OC_72h_KA_4 |
| OC_72h_CTRL_3 |
| OC_72h_KA_3 |
| OC_24h_CTRL_1 |
| OC_24h_CTRL_2 |
| OC_24h_CTRL_3 |
| OC_24h_KA_3 |
| OC_5h_CTRL_2 |
| OC_5h_KA_2 |
| OC_5h_KA_1 |
| OC_5h_CTRL_1 |
| OC_5h_KA_3 |
| OC_5h_CTRL_3 |
| OC_24h_KA_4 |
| OC_24h_CTRL_4 |
| OC_5h_KA_4 |
